# Survey radiography and computerized tomography imaging of the thorax in female dogs with mammary tumors

**DOI:** 10.1186/1751-0147-52-20

**Published:** 2010-03-09

**Authors:** Carolina C Otoni, Sheila C Rahal, Luiz C Vulcano, Sérgio M Ribeiro, Khadije Hette, Tatiana Giordano, Danuta P Doiche, Renée L Amorim

**Affiliations:** 1 São Paulo State University (Unesp), Department of Veterinary Surgery and Anesthesiology, School of Veterinary Medicine and Animal Science, Botucatu, SP, Brazil; 2 Unesp, Department of Animal Reproduction and Radiology, School of Veterinary Medicine and Animal Science, Botucatu, SP, Brazil; 3 Unesp, Department of Tropical Diseases and Diagnostic Imaging, Botucatu Medical School, Botucatu, SP, Brazil; 4 Unesp, Department of Veterinary Clinical Sciences, School of Veterinary Medicine and Animal Science, Botucatu, SP, Brazil

## Abstract

**Background:**

Accurate early diagnosis of lung metastases is important for establishing therapeutic measures. Therefore, the present study aimed to compare survey thoracic radiographs and computerized tomography (CT) scans to specifically identify lung metastases in female dogs with mammary tumors.

**Methods:**

Twenty-one female dogs, weighing 3 to 34 kg and aged from 5 years to 14 years and 10 months, with mammary tumors were studied. In all dogs before the imaging examinations, fine-needle aspiration cytology of the mammary tumors was performed to confirm the diagnosis. Three-view thoracic radiographs were accomplished: right lateral, left lateral and ventrodorsal views. Sequential transverse images of the thorax were acquired on a spiral Scanner, before and after intravenous bolus injection of nonionic iodine contrast. Soft-tissue and lung windows were applied. All the mammary tumors were surgically removed and examined histologically.

**Results:**

The correlation between the cytological and histological results regarding presence of malignancy was observed in only 17 cases. In radiographic examinations, no dog displayed signs of lung metastases or thorax chest lesions. CT detected lung metastasis in two cases, while small areas of lung atelectasis located peripherally were found in 28.57% of the dogs.

**Conclusion:**

In this study population, spiral CT showed higher sensitivity than chest radiographies to detect lung metastasis; this indicates that CT should be performed on all female dogs with malignant mammary tumors.

## Background

Mammary tumors constitute the most frequent neoplastic disease in female dogs [1,2]. The disease etiology has not yet been totally elucidated, but there are indications of a hormonal dependence because the incidence of tumors is reduced by using early ovariohysterectomy, with better results when the procedure is performed before the first estrus [1-3]. In addition, dogs with mammary gland carcinoma spayed less than 2 years before tumor surgery live longer than dogs spayed earlier in relation to such surgery [4].

Approximately 35% to 50% of all mammary tumors in female dogs are considered malignant by histological examinations [2,3]. The clinical stage, tumor size and ovariohysterectomy status are prognostic factors for dog survival after surgery to treat malignant mammary tumors [5]. Furthermore, these tumors may disseminate through the lymphatic and blood vessel routes to the regional lymph nodes and lungs [1,3]. Therefore, the accurate and early diagnosis of lung metastases is of considerable importance in the establishment of therapeutic measures, and approximately 25% and 50% of the female dogs with malignant mammary tumors already present them at the moment of the physical examination [3].

Among the imaging methods reported as frequently used for lung metastases identification are radiographic examinations, magnetic resonance and computerized tomography (CT) [3,6-9]. Radiographically, lung metastasis can be characterized by well-defined nodules, poorly demarcated nodules or as pleural effusion without any evidence of lung lesions [2]. Although CT is considered a more sensitive method than radiography for detecting lung metastases, false-positive or false-negative results may occur [8,10,11]. Therefore, various types of CT instruments are constantly being developed to obtain higher accuracy and potency. Furthermore, a short exposure time is very important to minimize the effects of cardiovascular and respiratory motion [12].

The present study aimed to compare survey thoracic radiographs and computerized tomography (CT) scans in relation to their ability to specifically identify lung metastases in female dogs with mammary tumors.

## Materials and methods

Twenty-one female dogs, weighing 3 to 34 kg and aged from 5 years to 14 years and 10 months (average of 9 years old and 5 months), with mammary tumors were utilized (Table 1). The time between tumor identification and surgical removal varied from 2 months to 1 year. Two dogs (Cases 6 and 19) had been submitted 3 months previously for regional mastectomy based on the lymphatic drainage in the other mammary chain. Four female dogs (Cases 1, 3, 6 and 21) had already been spayed. Before the imaging examinations in all female dogs, fine-needle aspiration cytology of the mammary tumors was performed to confirm the diagnosis. Only those with malignant mammary tumors were included in the study. The size of the primary tumor was classified according to maximum diameter as follows: T1 < 3 cm, T2 3-5 cm and T3 > 5 cm (Table 1). Laboratory tests, including a complete blood cell, urinalysis and chemistry panel, were carried out to detect any metabolic alterations that could contraindicate the procedures.

**Table 1:** Description of female dogs with mammary tumors; mammary glands with tumor and tumor size; fine-needle aspiration cytology and histological diagnosis of the mammary tumors.

Dog description	Mammary glands with tumor and tumor size	Fine-needle aspiration cytology	Time between tumor identification and surgical removal	Histological analysis
Case 1 34 kg-13.8-year-old German Shepherd (10 y spayed)	Left CrT and CT (T2 - ulcerated)	mammary carcinoma	2 mo	mammary carcinoma
**Case 2** **8 kg-11.10-year-old crossbred** **(intact)**	**Left CrA (T1)**	**Left CrA - adenoma mammary**	**9 mo**	**Left CrA - mammary carcinoma**
Case 3 13.4 kg-10.7-year-old crossbred (7 y spayed)	Right I (T2 - ulcerated) Left CA (T1)	Right I - mammary carcinoma; Left CA inflammatory process	3 mo	Right I - mammary carcinoma and regional lymph node metastases; Left CA -trichoepithelioma
**Case 4** **11.4 kg-9-year Poodle** **(intact)**	**Left CrA, CA and I (T2); Right CA (T1)**	**Left CrA, CA and I - secretory mammary carcinoma** **Right CA - absent neoplasia**	**9 mo**	**Left CrA, CA and I - mammary adenoma;** **Right CA - absent neoplasia**
Case 5 9.1 kg-9.4-year-old crossbred (intact)	Right CA (T1)	mammary carcinoma	3 mo	mammary carcinoma
**Case 6** **3 kg-5.8-year-old Yorkshire** **(3 mo spayed)**	**Right CrT (T1)**	**mammary carcinoma**	**9 mo**	**mammary carcinoma**
Case 7 3.5 kg-7-year-old crossbred (intact)	Left CT and CrA (T1); Right CrA (T1)	Right CrA and Left CrA - malignant mixed tumor; Left CT - epidermal cyst	7 mo	Right CrA and Left CrA - malignant mixed tumor; Left CT - epidermal cyst
**Case 8** **13 kg-9-year-old Poodle** **(intact)**	**All Left mammary glands (T2); Right CrA and I (T2)**	**Left mammary glands - mammary carcinoma;** **Right CrA and I - malignant mixed tumor**	**7 mo**	**Left mammary glands - mammary carcinoma;** **Right CrA and I -adenocarcinoma**
Case 9 7 kg-8.6-year-old crossbred (intact)	Left I (T1)	adenocarcinoma	1 y	benign mixed tumor
**Case 10** **6.5 kg-8-year-old crossbred** **(intact)**	**Right CA and I (T1)**	**Right CA and I - malignant mixed tumor**	**3 mo**	**Right CA and I -malignant mixed tumor**
Case 11 22.6 kg-6.8-year-old Cocker Spaniel (intact)	Right I (T1)	mammary carcinoma	1 y	mammary carcinoma
**Case 12** **5.8 kg-9-year-old Poodle (intact)**	**Right CT (T1)**	**mammary carcinoma**	**3 mo**	**mammary carcinoma**
Case 13 16.7 kg-6.3-year-old Dachshund (intact)	Left CT (T3)	mammary carcinoma	4 mo	mammary carcinoma
**Case 14** **31 kg-8.5-year-old Boxer** **(intact)**	**Right CA (T3)**	**mammary carcinoma**	**2 mo**	**malignant mixed tumor**
Case 15 14.1 kg-14.7-year-old Poodle (intact)	Right CT (T3)	mammary carcinoma	7 mo	papillary cystadenocarcinoma
**Case 16** **3.6 kg-14.10-year-old crossbred** **(intact)**	**Right CA (T1), Right I (T2);** **Left CrA (T1), Left I (T1)**	**Left CrA and I - malignant mixed tumor; Right CA -** **secretory adenoma**	**1 y**	**malignant mixed tumor**
Case 17 21.5 kg-14-year-old crossbred (intact)	Right CrA and CA (T3 - ulcerated); left I (T2)	malignant mixed tumor	3 mo	malignant mixed tumor
**Case 18** **3.4 kg-5-year-old crossbred** **(intact)**	**Left CA (T1)**	**mammary carcinoma**	**9 mo**	**mammary carcinoma**
Case 19 11.2 kg-8-year-old Cocker Spaniel (intact)	Right I (T1)	mammary carcinoma	1 y	mammary tubular carcinoma
**Case 20** **27 kg-6.5-year-old Akita** **(intact)**	**Right CA (T3)**	**mammary adenoma**	**4 mo**	**mammary adenocarcinoma**
Case 21 10.7 kg-13-year-old crossbred (8 y spayed and received contraceptive)	Right CrA (T2); Left CrA (T1)	malignant mixed tumor	2 y	malignant mixed tumor

Cranial thoracic - CrT; Caudal thoracic - CT; Cranial abdominal - CrA; Caudal Abdominal - CA; Inguinal -I; T1 - tumor < 3 cm maximum diameter, T2 - tumor 3-5 cm maximum diameter, T3 - tumor > 5 cm maximum diameter.

### Preoperative Radiographic and CT studies

Thoracic radiographs were taken from three views: right lateral, left lateral and ventrodorsal. Green-sensitive film (Kodak) and cassettes with screens were used. A focus-film distance of 90 cm was used with an exposure of 50-70 kV and 3.2-5.0 mAs for the lateral view and 45-65 kV and 3.2-5.0 mAs for the ventrodorsal view, according to the size of each dog. The radiographs were done at peak inspiration. An X-ray unit type TUR D800-4, 125 kVp/500 mA capacity, equipped with Potter-Bucky grid was used. The film was processed with an automatic processor (Macrotec MX2). To maintain the quality control of the radiographs, in both ventrodorsal and lateral views the thorax should not be rotated. In addition, the sternum and vertebra should be superimposed on the ventrodorsal view, and little or no contact between the diaphragm and the heart should be seen on the lateral view. The technical quality of the film was considered satisfactory if there was a clear contrast among the structures - pulmonary vessels, heart and air-filled lungs. The thoracic radiographs were evaluated starting from cardiac silhouette, trachea, mediastinum and pleural space, and ending at the lungs. Radiographs were classified as either positive or negative for pulmonary metastases, and other pulmonary diseases. In addition, the heart aspect, the presence of mediastinal masses, and thoracic wall alteration were evaluated.

To perform CT examinations the female dogs were premedicated with acepromazine 0.03 mg/kg, IM, and morphine 0.5 mg/kg, IM. After approximately 10 minutes, dissociative anesthesia was induced and maintained with ketamine 3 mg/kg and diazepam 0.5 mg/kg, administered intravenously, with the dogs positioned in sternal recumbency. The lungs were not mechanically inflated for imaging. The female dogs were placed in dorsal recumbency with the forelimbs pulled cranially and the hind limbs caudally. Sequential transverse images from the first thoracic vertebra to lumbar diaphragmatic recess were acquired on a spiral Scanner (Shimadzu SCT-7800CT). After plain CT study, a contrast study was done using 2 ml/kg, IV, bolus injection of nonionic iodine contrast agent (iohexol or meglumine diatrizoate). The scanning parameters were 120 kVp, 150-180 mA, 2.0-3.0 mm collimation, 2:0 pitch, and 1-second scan time. The field of view was 168-409 mm according to the dog's size, while the matrix was 512 × 512 mm. The software used to read the CT-images was 2.1.2 eFilm (TM) Lite (TM) (MERGE Healthcare). The study was carried out using soft-tissue and lung windows, and it was started from the mediastinum to chest wall in order to detect lesions. The plain CT images were compared with the contrast images. CT images were classified as either positive or negative for pulmonary metastases. When nodules were present, their diameters were found by means of a measuring tool from an image analysis software package. Three experienced radiologists performed blind evaluation of the radiographs and CT.

### Surgical procedures, and Histology

After ovariohysterectomy, either a unilateral, bilateral or regional mastectomy was performed, based on an assessment of lymphatic drainage. Lumpectomy was used specifically in cases when other benign masses were present.

The mammary tumors removed at surgery were immediately fixed in 10% buffered formalin. Semi-serial sections, 4-μm-thick, were obtained and stained with hematoxylin and eosin. The histological results were compared with those obtained previously by fine-needle aspiration cytology.

## Results

Ovariohysterectomy was performed on 13 female dogs, and in four cases (Cases. 15 and 17-19), the owner did not authorize this procedure. The correlation between the cytological and histological results regarding presence of malignancy was observed in 17 cases. However, in two cases (Cases. 4 and 9) the fine-needle aspiration cytology suggested malignant tumor and histological analysis showed a benign process, and in another two cases (Cases. 2 and 20) the fine-needle aspiration cytology suggested benign tumor and histological analysis showed a malignant tumor (Table 1).

**Figure 1:**
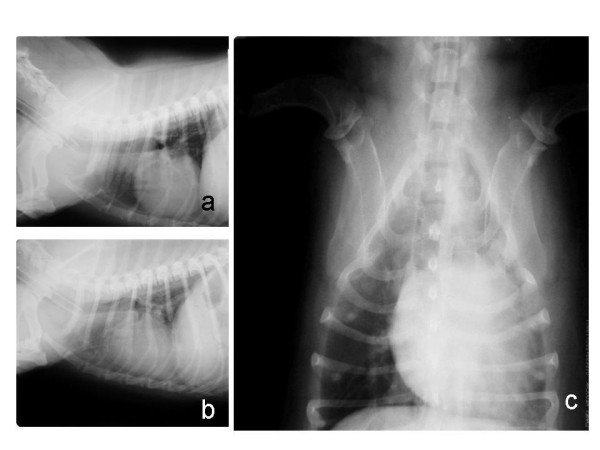
**Right lateral (a), left lateral (b), and ventrodorsal (c) radiographic views of the thorax of a female dog (Case 13) with mammary carcinoma**. No signs of metastases were observed in the lungs.

**Figure 2:**
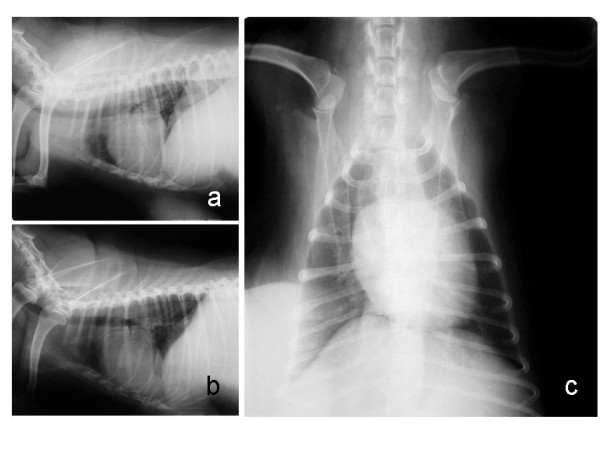
**Right lateral (a), left lateral (b), and ventrodorsal (c) radiographic views of the thorax of a female dog (Case 15) with mammary papillary cystadenocarcinoma**. No signs of metastases were observed in the lungs.

**Figure 3:**
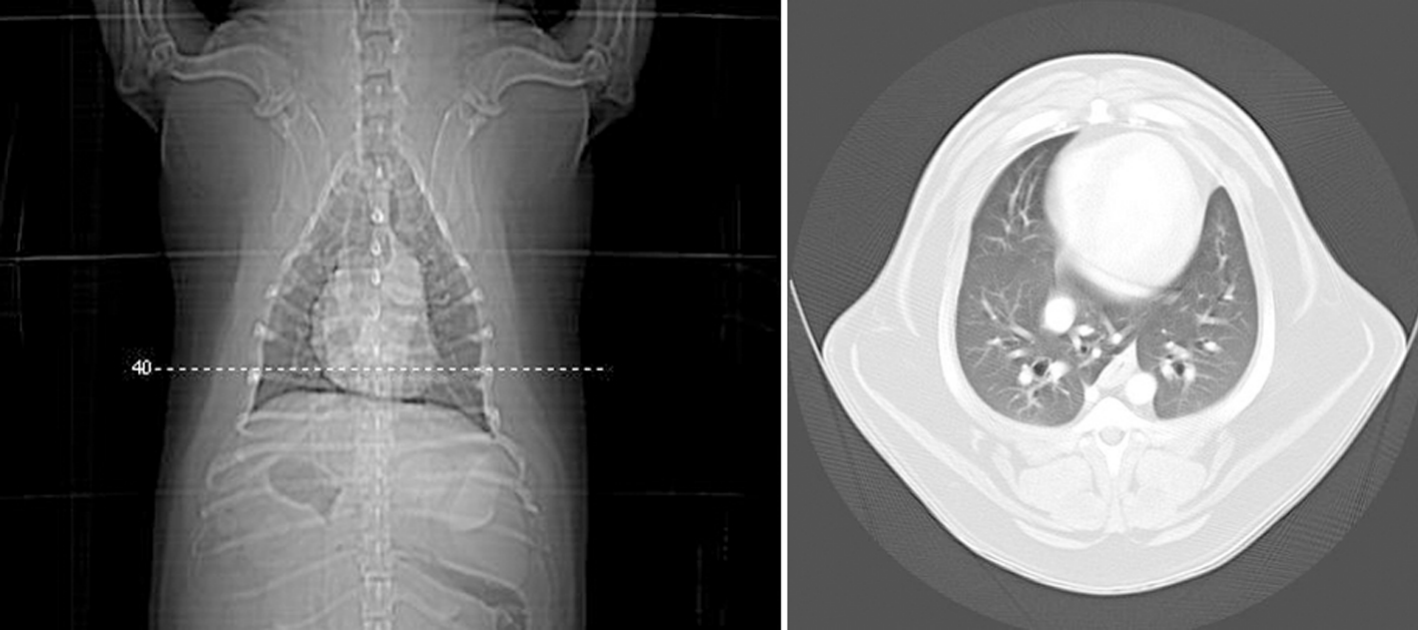
**Transverse CT views at the level of the thoracic diaphragmatic lobes of a female dog (Case 13) with mammary carcinoma**. No signs of metastases were observed in the lungs.

**Figure 4:**
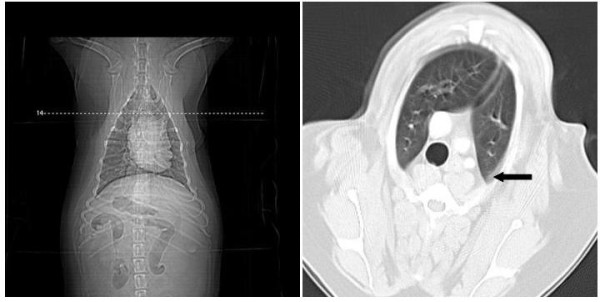
**Transverse CT views of the thorax of a female dog (Case 11) showing small area of atelectasis (arrow)**.

**Figure 5:**
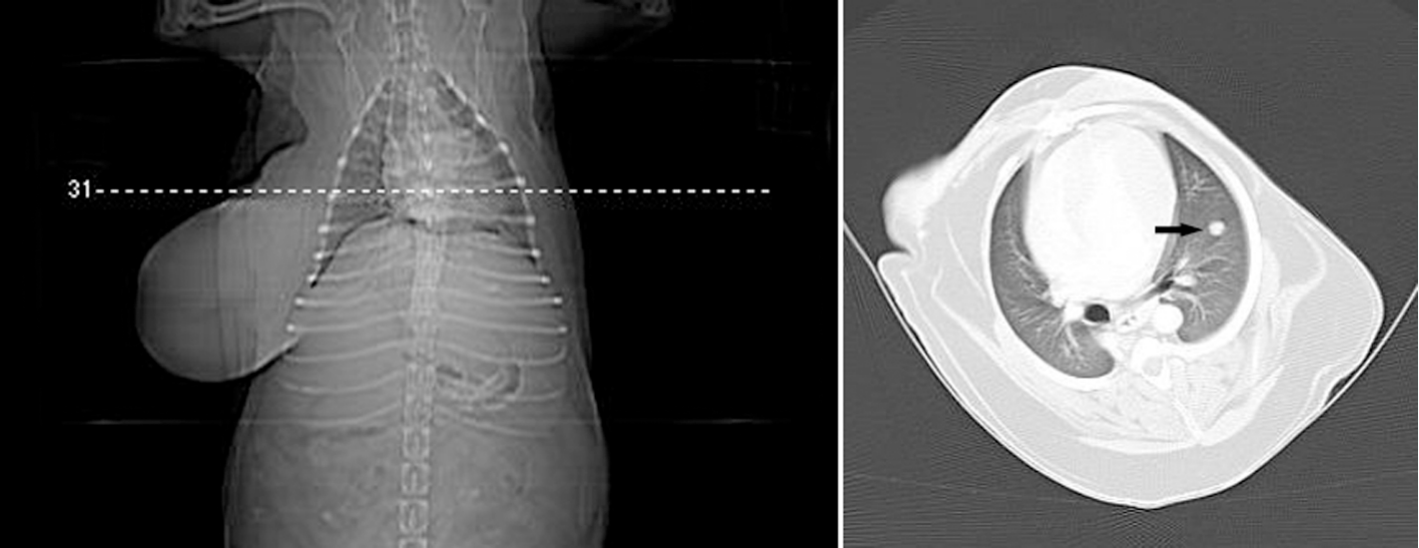
**Transverse CT views at the level of the right medial lobe and left apical lobe and thorax of a female dog (Case 15) with mammary papillary cystadenocarcinoma**. Observe 0.7 cm nodule in maximum dimension located on the medial portion of the left caudal apical lobe (arrow). Note tumor present in the right caudal thoracic region.

**Figure 6:**
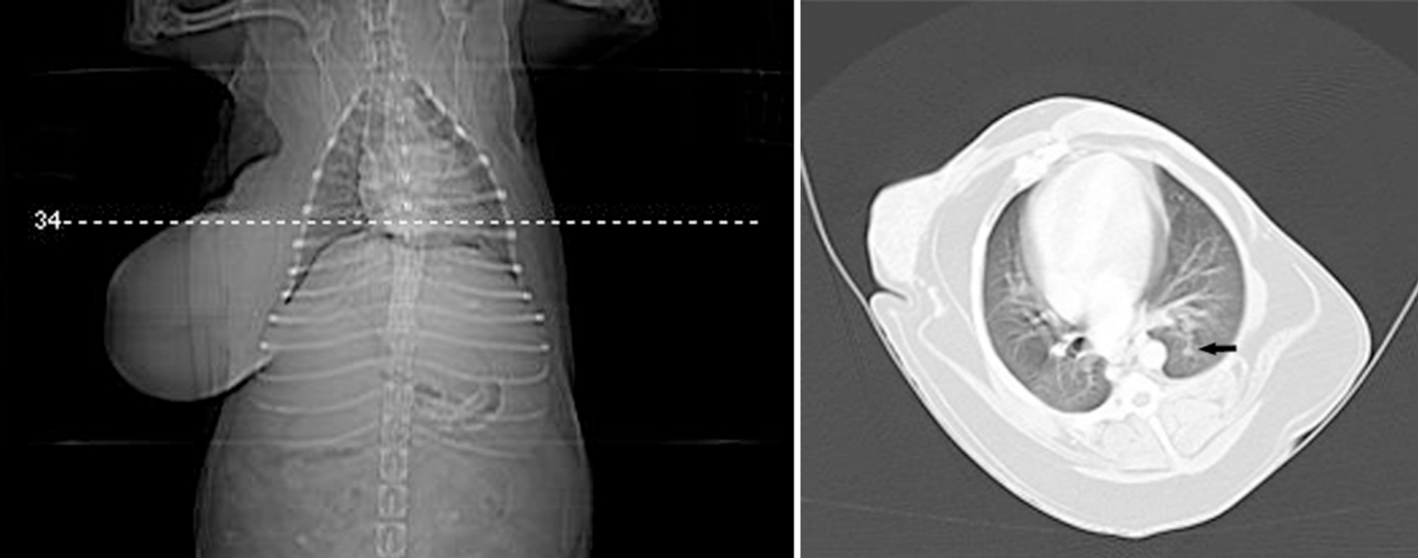
**Transverse CT views at the level of the right medial lobe and left diaphragmatic thorax of the female dog (Case 15) with mammary papillary cystadenocarcinoma**. Observe 0.4 cm nodule in maximum dimension located on the medial portion of the left diaphragmatic lobe showing signal of nutrient vessel (arrow).

**Figure 7:**
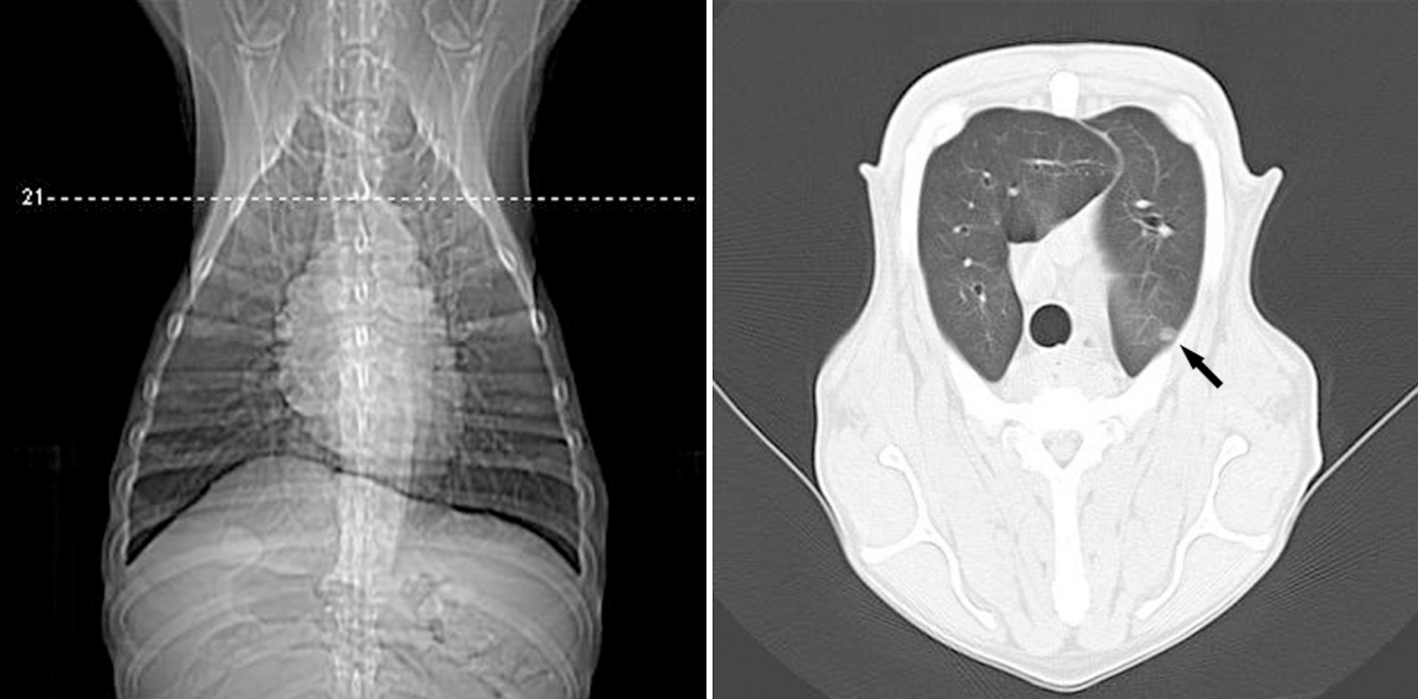
**Transverse CT views at the level of the right apical lobe and left thorax of a female dog (Case 17) with malignant mixed mammary tumor**. Observe 0.6 cm nodule in maximum dimension located on the left posterior cranial apical lobe (arrow).

**Figure 8:**
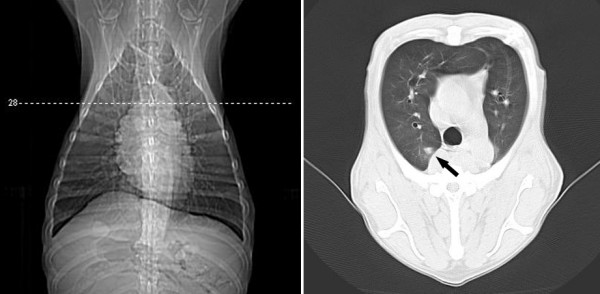
**Transverse CT views at the level of the right apical lobe and left thorax of a female dog (Case 17) with malignant mixed mammary tumor**. Observe 0.9 cm nodule in maximum dimension with irregular outline, clear-cut limits and surrounded by ground-glass opacity, located on the posterior portion of the right apical lobe (arrow).

Radiographic examinations showed no signs of lung metastases or thorax chest lesions in any dog (Fig. 1 and Fig. 2). CT examination found no alteration in the chest wall or mediastinum (Fig. 3). However, lung metastasis was found in two cases (Cases 15 and 17), and lung atelectatic areas were detected in 28.57% of the dogs (Cases. 3, 8, 11, 14, 17 and 20). The atelectatic areas were small focal infiltrates and located peripherally (Fig. 4). Case 15 had five solid nodules with regular outline that was well-defined at the left lung parenchyma, thus characteristic for a lung metastasis. The larger nodule measured 0.7 cm in maximum dimension and was located on the medial portion of the left caudal apical lobe (Fig. 5). Another nodule, measuring 0.4 cm in maximum dimension, located on the medial portion of the left diaphragmatic lobe presented the signal of a nutrient vessel (Fig. 6). Case 17 had two nodules, one 0.6 cm nodule in maximum dimension located on the left posterior cranial apical lobe (Fig. 7) and another measuring 0.9 cm in maximum dimension with irregular outline, clear-cut limits and surrounded by ground-glass opacity (Fig. 8).

## Discussion

Carcinoma, sarcoma, and malignant mixed tumor are considered the most common malignant mammary tumors [2,3], with the latter occurring most frequently [13]. In the present study, histological examinations showed 13 mammary carcinomas and 6 malignant mixed tumors. However, in two female dogs, histology revealed benign tumors (mammary adenoma and benign mixed tumor) that were found to be malignant by fine-needle aspiration cytology, and other two cases the aspiration cytology suggested malignant tumor and histological analysis showed a benign process. Some authors have discouraged the use of fine-needle aspiration cytology due to its insensitivity in differentiating between malignant and benign mammary tumors [2].

Evaluation of the lungs by imaging studies is necessary in all cases of mammary tumors. Because of the high blood flow and capillary network that provoke slower circulation, the lungs are in addition to the regional lymph nodes the most common sites for metastases [1,14,15]. The use of two or three radiographic views to evaluate lung diseases in dogs remains controversial [6,16]. The present study employed three views, because eliminating one radiographic view may influence the diagnosis in 12-15% of the patients [17]. However, three views were not sufficient to detect the metastases, showing that radiography is less sensitive than CT [10,11]. In 18 dogs with pulmonary metastatic neoplasia, only 9% of the pulmonary nodules detected in CT were observed on radiographs. Besides, pulmonary nodules were identified in a greater number of lung lobes by CT than radiographs [8].

The dogs were maintained in spontaneous breathing during CT examination due to the type of anesthesia used. However, the images obtained during the apneic period and at the inspiration peak are considered better to minimize the breathing artifact at the tumor location and size [18,19]. Some authors have used intermittent positive-pressure ventilation to decrease motion artifacts [8,20]. On the other hand, in a study of dogs with metastatic osteosarcoma using spiral CT with a collimation of 5 mm and pitch of 2, no significant difference was observed between normal resting respiration and holding of breath [21].

Technical modifications may be performed to obtain an optimal lung CT [22]. In a study of two dogs with pulmonary nodules evaluated by helical CT, the suggested protocol included a narrow collimation (3-5 mm according to the dog's size), pitch of 2 and interval reconstruction of 1 [23]. A similar protocol was used in the present study. CT examinations with intravenous contrast injection were performed to obtain a better definition of the vessels and to differentiate between normal and abnormal vascular structures [18,24]. In addition, soft-tissue and lung windows were utilized to enable evaluation of chest wall, and lung parenchyma [20,18].

The dogs were positioned in dorsal recumbency, although some authors suggest that sternal recumbency provides better quality and absence of artifacts [18]. Atelectatic areas were observed by CT in 28.57% of the cases, but these areas are probably associated with recumbency and gravitational stasis [24] or anesthetic procedure [25], since they are not presented radiographically. Another cause of atelectasia is the transport of the patient under general anesthesia to the CT gantry in lateral recumbency [26]. In a study of dogs with metastatic neoplasia positioned in ventral recumbency, when the initial image showed ventral atelectasis, the CT was repeated with the patient in dorsal recumbency to allow better inflation of these lung regions [8]. The atelectatic areas may show variable CT appearance such as focal interstitial infiltrates, alveolar infiltrates or complete lobar collapse [19,22]. In the present study most of these areas were peripheral small focal infiltrates and apparently did not influence the results.

CT allowed detection of metastases in two cases in the present study, thus showing greater precision than the plain radiographic examination that it is considered less sensitive for detection of small lesions [7,8,16,27]. In a retrospective study of 18 dogs with pulmonary metastatic neoplasia, the smallest size to detect pulmonary nodules on CT images was 1 mm, compared with 7-9 mm on radiographs [8]. However, in a study of four dogs presenting lung metastases of osteosarcoma confirmed by plain thoracic radiographic examination, the spiral CT did not detect 32 metastases measuring ≤1 cm in diameter [10]. Thus, the low number of lung metastases observed in the present study may be underestimated due to the limitation of the imaging examinations used. Probably this fact is associated with the citation that most female dogs with malignant tumors without metastases at the moment of the surgery will have died or will have been euthanized on account of problems associated with the tumor within 1 or 2 years [3].

Case no. 17 had five solid nodules with regular outline and was well-defined at the lung parenchyma, which characterizes lung metastases. This standard of lung metastases is compatible with the occurrence of the hematogenous dissemination, which can occur as single, multiple or propagated nodules, with generally random distribution. However, the metastatic nodules are generally circular with regular contour, especially in more aggressive lesions, such as those of sarcomatous origin [28]. Furthermore, the typical metastatic carcinoma usually occurs as numerous small circular lesions [29].

Case 15 had two pulmonary nodules, one of which presented an irregular outline, clear-cut limits and was surrounded by ground-glass opacity. The metastatic nodules may have irregular or lobular contour [28].

## Conclusions

In this study population, spiral CT showed higher sensitivity than chest radiographies to detect lung metastasis; this indicates that CT should be performed on all female dogs with malignant mammary tumors.

## Competing interests

The authors declare that they have no competing interests.

## Authors' contributions

CCO, SCR and KH participated in the study design, surgical procedures, and drafted the manuscript. LCV, SMR and DPD interpreted the radiographic and CT examinations. TG performed the anesthesia procedures. RLA performed the microscopic examinations. All authors read and approved the final manuscript.
